# Asymmetron: a toolkit for the identification of strand asymmetry patterns in biological sequences

**DOI:** 10.1093/nar/gkaa1052

**Published:** 2020-11-19

**Authors:** Ilias Georgakopoulos-Soares, Ioannis Mouratidis, Guillermo E Parada, Navneet Matharu, Martin Hemberg, Nadav Ahituv

**Affiliations:** Department of Bioengineering and Therapeutic Sciences, University of California San Francisco, San Francisco, CA, USA; Institute for Human Genetics, University of California San Francisco, San Francisco, CA, USA; Aristotle University of Thessaloniki, Department of Mathematics, Thessaloniki, GR, Greece; Wellcome Sanger Institute, Wellcome Genome Campus, Hinxton, CB10 1SA, UK; Wellcome Trust Cancer Research UK Gurdon Institute, University of Cambridge, Tennis Court Road, Cambridge CB2 1QN, UK; Department of Bioengineering and Therapeutic Sciences, University of California San Francisco, San Francisco, CA, USA; Institute for Human Genetics, University of California San Francisco, San Francisco, CA, USA; Innovative Genomics Institute, University of California San Francisco, San Francisco, CA, USA; Wellcome Sanger Institute, Wellcome Genome Campus, Hinxton, CB10 1SA, UK; Wellcome Trust Cancer Research UK Gurdon Institute, University of Cambridge, Tennis Court Road, Cambridge CB2 1QN, UK; Department of Bioengineering and Therapeutic Sciences, University of California San Francisco, San Francisco, CA, USA; Institute for Human Genetics, University of California San Francisco, San Francisco, CA, USA

## Abstract

DNA strand asymmetries can have a major effect on several biological functions, including replication, transcription and transcription factor binding. As such, DNA strand asymmetries and mutational strand bias can provide information about biological function. However, a versatile tool to explore this does not exist. Here, we present Asymmetron, a user-friendly computational tool that performs statistical analysis and visualizations for the evaluation of strand asymmetries. Asymmetron takes as input DNA features provided with strand annotation and outputs strand asymmetries for consecutive occurrences of a single DNA feature or between pairs of features. We illustrate the use of Asymmetron by identifying transcriptional and replicative strand asymmetries of germline structural variant breakpoints. We also show that the orientation of the binding sites of 45% of human transcription factors analyzed have a significant DNA strand bias in transcribed regions, that is also corroborated in ChIP-seq analyses, and is likely associated with transcription. In summary, we provide a novel tool to assess DNA strand asymmetries and show how it can be used to derive new insights across a variety of biological disciplines.

## INTRODUCTION

Even though the DNA double helix is a symmetric structure, many biological processes such as replication, transcription and transcription factor binding are directional. The directionality of these processes results in the inhomogeneous distribution of genomic sequences relative to the two complementary DNA strands. Reflecting directionality biases, strong compositional strand asymmetries have been observed across the entire tree of life, ranging all the way from viral to eukaryotic genomes. This bias has been ascribed to replication origins and transcription initiation sites in all these organisms (1–6). In double-stranded DNA viruses, a GC-skew, which measures the asymmetry in the distribution of Gs and Cs in the two strands, has been observed between the leading and lagging strands (7). In prokaryotic genomes, genes are more frequently observed in the leading orientation, a phenomenon that is more pronounced for essential genes (8). This asymmetry is in accordance with evidence suggesting that genes in the lagging strand accumulate an excess of mutations relative to those in the leading orientation (9). In mammals, the testis expresses the highest number of genes relative to any other tissue. This mechanism safeguards the germline DNA integrity through reduced mutations at the transcribed strand as a result of transcription-coupled repair and in turn leads to reduced population diversity at those sequences (10).

DNA mutations can be oriented relative to transcription and replication, using as reference the template/non-template and leading/lagging strands, respectively. If the reference nucleotide or motif at the site of the mutation is found more frequently in one strand relative to the other, following correction for background strand preferences, it indicates a mutational strand asymmetry. This mutational strand imbalance can have a major impact on disease, development and evolution. For example, the transcription-coupled repair pathway preferentially repairs DNA damage at the template strand, as it can otherwise impede the RNA polymerase progression (11). In lung cancer, tobacco-related carcinogens form bulky adducts at guanines and their preferential repair at the template strand of expressed genes results in mutational imbalance of G>T substitutions (12). The apolipoprotein B mRNA editing enzyme, catalytic polypeptide-like (APOBEC) is a cytidine deaminase involved in antiviral defense. However, off-target APOBEC-associated mutagenesis in the human genome is often observed in cancer cells and more frequently targets cytosines on the lagging replicative strand (13,14). Transcriptional and replicative strand asymmetries have also been characterized in relationship with gene expression levels and replication timing, providing further mechanistic insights (13–15).

Sequences that are non-palindromic can be oriented relative to one another. A pair of motifs can be on the same or opposite strands and if they are on opposite strands they can be in convergent (facing each other) or divergent (facing away from each other) orientations. Examples include many transcription factor binding sites. One of the most notable is the CCCTC-binding factor (*CTCF*), the motif orientation of which dictates chromatin looping and three dimensional genome topology (16). Another noteworthy example is the Ying-Yang 1 (*YY1*) transcription factor, whose motif orientation in the *c-Fos* promoter reverses the expression of the downstream gene, therefore acting either as an activator or a repressor depending on the genomic orientation of its motif relative to the transcriptional direction (17). In transcription factor heterodimers, the orientation of the individual motifs can also influence binding and expression levels (18,19). Other cases of strand asymmetries include endogenous repetitive elements with preferences in their orientation relative to each other and relative to transcriptional and replicative direction, which could influence their jumping activity (20,21) or the relative orientation of genes and proximal long non-coding RNAs that can regulate the expression of each other. In particular, there is evidence that antisense transcripts can form self-regulatory circuits with the target gene (22).

**Figure 1.**
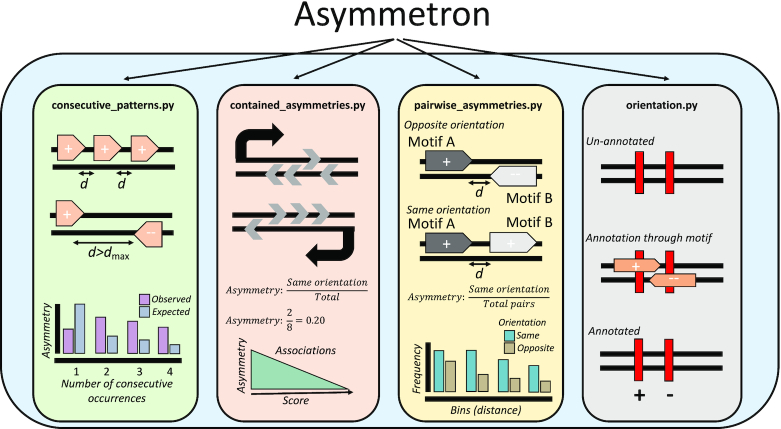
Graphical depiction of Asymmetron functionalities. The Asymmetron toolkit is composed of four functions that enable the estimation of strand asymmetries within and between BED file datasets. The consecutive_patterns.py function enables the identification of patterns within consecutive occurrences of a feature. The contained_asymmetries.py function calculates the strand asymmetries of a feature of interest (motifs) contained within another feature (regions). The pairwise_asymmetries.py function estimates the orientation bias between two features that are in proximity to each other. The orientation.py function orients an un-annotated feature relative to another overlapping feature that has strand annotation; it is integrated within all three other functions and can also run independently.

Characterization of strand asymmetries can thus allow for the identification of novel DNA elements, improve our understanding regarding their interactions with one another, and advance our knowledge of the underlying processes in mutagenesis and evolution. To date, there is no versatile tool to perform analysis of strand asymmetries across biological problems. Here, we introduce Asymmetron, a novel, multi-purpose computational toolkit that systematically characterizes strand asymmetry patterns in nucleotide sequences. Asymmetron is composed of four functions (Figure 1), the first being ‘*consecutive_patterns.py*’ which finds strand patterns within consecutive occurrences of a single genomic element, ‘*contained_asymmetries.py*’ is used for pairs of genomic elements in which one is contained within the other, and ‘*pairwise_asymmetries.py*’ which finds asymmetries between the pairs of proximal genomic elements. The fourth function, ‘*orientation.py’* assigns strand asymmetries from one genomic element to another and can be used to orient features of interest such as Chromatin immunoprecipitation followed by sequencing (ChIP-seq) peaks or mutations relative to strand-assigned genomic sequences of interest. Using Asymmetron we show that germline structural variant breakpoints can be oriented relative to transposable elements and find transcriptional and replicative strand asymmetries in them, suggesting transposable element activity in the germline. We also provide evidence that the orientation of many transcription factor binding sites (TFBSs) is highly biased across promoters and in transcribed regions and validate our findings by analyzing the orientation of TFBSs within ChIP-seq peaks.

## MATERIALS AND METHODS

Asymmetron enables versatile genomic investigations of strand asymmetry patterns across different biological problems. It is a Python-based toolkit and its core BED-formatted file comparison functions use the package Pybedtools (23). Asymmetron provides support for three types of analyses: (i) consecutive strand asymmetry estimation in a single file with strand annotation; (ii) strand asymmetry estimation of strand-assigned motifs within strand-assigned regions; (iii) strand asymmetry estimation between two strand-assigned motifs in proximity or overlapping each other. A fourth function performs the strand assignment of an unassigned feature based on another overlapping feature, thereby enabling the strand asymmetry analysis of the first (Figure 1).

Let us define the alphabet $L\ = \ \{ {A,\ T,\ C,\ G} \}$. DNA can be represented by a pair of sequences ${A_n} = {a_1}\ {a_2}{a_3} \ldots {a_n}$, where ${a_i} \in L\ for\ i\ = \ 1,2,3 \ldots n$ and ${B_n} = {b_1}\ {b_2}{b_3} \ldots {b_n}$, its complement strand, where ${b_i} = \ A\,if\,{a_i} = T,\,\,{b_i} = {\rm{ }}G$ if ${a_i} = {\rm{ }}C,\,{b_i}\ = {\rm{ }}C{\rm{ }}if\ {a_i} = {\rm{ }}G$ and ${b_i} = {\rm{ }}T$ if ${a_i} = {\rm{ }}A$. Because of the directionality of the two strands, we read *B_n_* from right to left, e.g. if *B_n_* = AG**GCT**, we will say that *B_n_* contains the motif TCG. Here, we use ‘motif’ to refer to a short sequence from the same alphabet that is of particular interest.

Analyses are often performed on genomic data, to extract all locations of a specific motif. In Asymmetron, we use these locations to estimate strand asymmetries through several types of analyses. We use these methods to evaluate strand asymmetries of non-palindromic sequences. To represent the locations of the motif in the genome, it is enough to save the chromosome, the index where the motif starts, the index where the motif ends as well as which strand the motif is found at. A common format used to store this information is a BED file, which, *inter alia*, saves the above mentioned information. In this format, the strand is represented with a + or − sign for *An* or *B_n_* respectively, which we will also use here. The information of a BED file relevant for our analyses can be represented as a set of vectors *S*, where in each vector chromosome is represented as *c*, start coordinate is represented as *s*, end coordinate as *e* sand sign as *r*.

The commands used to perform the analyses and the files can be found on the GitHub page (https://github.com/Ahituv-lab/Asymmetron). Asymmetron documentation, including a tutorial, several examples and description of all available options is available in http://asymmetron.readthedocs.io/.

### Consecutive strand asymmetry estimation for single motifs

**Figure 2.**
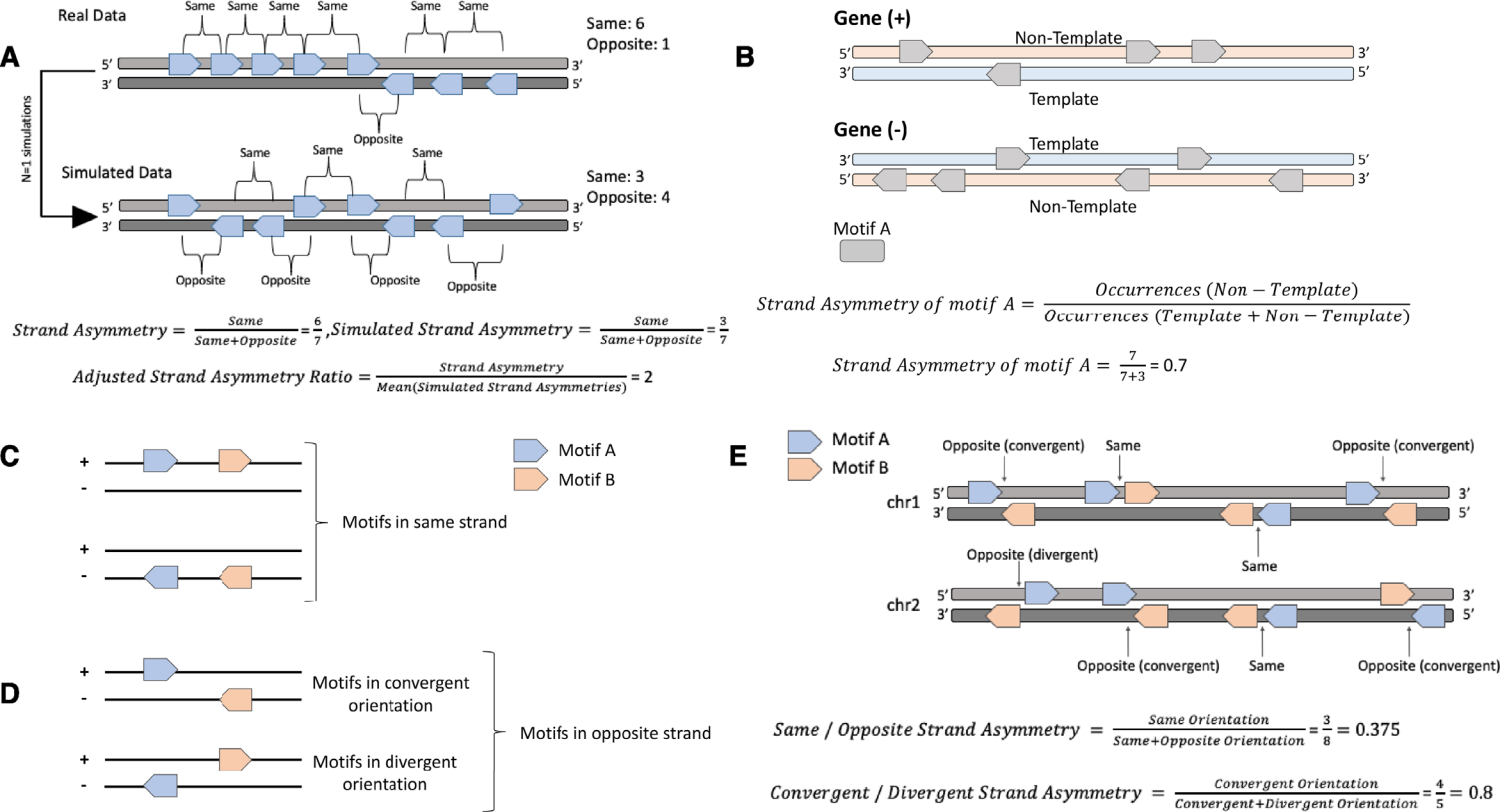
Schematic of strand asymmetry analyses across different scenarios. (**A**) Estimation of biases in the orientation patterns of consecutive occurrences of a motif relative to those found in the shuffled simulated data. Calculation of orientation patterns for consecutive motif occurrences is performed using the function *consecutive_patterns.py* function. In the presented example, there are seven motifs, six of them in the same orientation and one in the opposite orientation. We perform *N* simulations (in the schematic *N = 1*) and calculate the adjusted strand asymmetry ratio and empirical *P*-value. In this simple case, simulated strand asymmetry = 3/7 < strand asymmetry = 6/7, so the set of successes, as defined in the methods section, for which the simulated asymmetry is higher than the strand asymmetry has a cardinality of 0. This results in a trivial *P*-value of 1, as is to be expected from only a single simulation. (**B**) Estimation of transcriptional strand asymmetry of a motif in genic regions. Genes in both orientations are shown. Calculation of transcriptional strand asymmetries can be performed using the function *contained_asymmetries.py*. In the schematic, there are ten motifs distributed across two opposite oriented genes; the null hypothesis is that they are equally-likely to have either orientation relative to the gene direction. There are seven motifs in the non-template orientation resulting in $p - value\ = \ \mathop \sum \limits_{i\ = \ 0}^3 C( {10,i} ) \times {p^i} \times {q^{10 - i}} + \ \mathop \sum \limits_{i\ = \ 7}^{10} C( {10,i} ) \times {p^i} \times \ {q^{10 - i}} = \ 0.34$, calculated with the two-tailed binomial test. Motifs can occur in (**C**) same (++ and –) or (**D**) in opposite (+- and −+) strand orientations. The order of two same-type or different motifs is not taken into consideration because the double strand DNA molecule is bidirectional; nevertheless, if a third strand-oriented feature was included, their order would be another factor to account for. (**D**) For those motifs in opposite strands, they can be separated in convergent (+−) or divergent (-+) orientations. These orientations of motif pairs are specific to non-palindromic motifs. (**E**) Orientation of motif pairs and estimation of same/opposite and convergent / divergent strand asymmetry ratios using a miniature genome example of two chromosomes and several occurrences of two motifs in pairs. Calculation of the strand asymmetry for motif pairs is performed with the function *pairwise_asymmetries.py*. In the schematic, there are eight motif pairs, across the two chromosomes; the null hypothesis is that they are equally-likely to have same or opposite orientation and in the subset of opposite orientation cases, they are equally likely to be in convergent or divergent orientation. There are three motif pairs in same orientation, resulting in $p - value\ = \ \mathop \sum \limits_{i\ = \ 0}^3 C( {8,i} ) \times {p^i} \times {q^{8 - i}} + \ \mathop \sum \limits_{i\ = \ 5}^8 C( {8,i} ) \times {p^i} \times \ {q^{8 - i}} = \ 0.73$ and there are five motif pairs in opposite orientation resulting in $p - value\ = \ \mathop \sum \limits_{i\ = \ 0}^1 C( {1,i} ) \times {p^i} \times {q^{8 - i}} + \ \mathop \sum \limits_{i\ = \ 4}^5 C( {5,i} ) \times {p^i} \times \ {q^{5 - i}} = \ 0.38$, for same/opposite and convergent/divergent strand asymmetries, respectively, calculated with the two-tailed binomial test.

Nearby recurrences of a motif in the genome can have biological significance. To examine the patterns emerging from recurring motifs, we developed this function which allows the observation of consecutive occurrences of a motif. It analyzes whether there is an asymmetry in the number of times the motif appears in one strand versus the other (Figure 2A).

Let *S* be the vector representation of the input BED file. Let ${v_{( 1 )}},{v_{( 2 )}},{v_{( 3 )}}, \ldots ,{v_{( n )}}$ be the vectors of set *S* sorted first by chromosome *c* and then by start position *s*. We define the distance between two consecutive appearances of the motif in the same chromosome as $d\ ( {{v_{( i )}},{v_{( {i + 1} )}}} ) = \min \{ {0,\ {s_{( {i + 1} )}}\ --{e_{( i )}}} \}$. If $d\langle {{d_{min}}\ or\ d} \rangle {d_{max}}$ they are not considered consecutive for the purpose of this analysis. Let $C\ = \{ {c_1}\ ,{c_2}, \ldots ,{c_n}\}$ be a set consisting of sequences of characters ${c_i} = {c_{{i_1}}}\ {c_{{i_2}}} \ldots {c_{{i_k}}}$, where each character is the sign of an appearance of the motif that fit the previously mentioned criteria. We define *m* as the cardinality of the set $\{ ( {k,l} ):{c_{{k_l}}} = {c_{{k_{l + 1}}}}\ \}$, which represents all consecutive appearances of the motif on the same strand (both on *A_n_* or both on *B_n_* ). Similarly, *o* is defined as the cardinality of the set $\{ ( {k,l} ):{c_{{k_l}}} \ne {c_{{k_{l + 1}}}}\}$, which represents all consecutive appearances of the motif on opposite strands (one *A_n_* and the other on *B_n_*). The strand asymmetry ratio is defined as $r\ = \ m\ /( {m + o} )$, which represents the magnitude of consecutive orientation bias. We then run *N* simulations (default: *N* = 1000), randomly assigning a value (‘+’ or ‘−‘) to every ${c_{{i_k}}}$, while keeping the total number of ‘+’ and ‘-’ in *C* constant. Following the same procedure as above, the strand asymmetry ratio ${r_{si{m_j}}},{\rm{\ }}j\ = \ 1, \ldots ,N$ is calculated. The adjusted strand asymmetry ratio is then defined as the original strand asymmetry ratio *r* divided by the mean strand asymmetry ratio ${r_{sim}} = \frac{{\mathop \sum \nolimits_{j = 1}^N {r_{si{m_j}}}}}{N}$ across simulations. We define a success as ${r_{si{m_j}}} >r$. Let *L* be the number of successes. We use the cardinality of *L* to calculate the empirical *P*-value as follows:

$p - value\ = \ min( {1,2*\frac{{l + 1}}{{N + 1}}} )$, where $l\ = \ {\rm{min}}( {N - | L |,| L |} )$. We multiply by 2, to ensure that the *P*-value is not over-estimated, due to the two-tailed test.

The outputs of this tool include a table with the statistical evaluation of the asymmetry bias for each inputted pattern; BED files with statistically biased coordinates consecutively observed for each inputted pattern with an extra column having their estimated Bonferroni corrected *P*-value and barplot visualizations of the distribution of observed versus expected consecutive occurrences of each pattern and other relevant statistics. As an extension, the tool also offers the option to analyze custom patterns provided by the user.

### Strand asymmetry estimation between regions and overlapping motifs

The strand asymmetry between regions and overlapping motifs tool requires a set of strand-oriented BED-formatted files of the regions of interest and a set of strand-oriented BED-formatted motif files. The tool performs independently the analysis across pairs of region and motif files and measures the strand asymmetry scores for the motifs overlapping or contained in the regions (Figure 2B).

Let *S_1_, S_2_* be the set representation of two strand-annotated BED files. For each vector $({c_i},{s_i},{e_i},{r_i})$ in *S*_1_, this function will compare it to every vector $( {c_i^{\prime},\ s_i^{\prime},\ e_i^{\prime},\ r_i^{\prime}} )$ in *S_2_*. If $\exists k \in [{s^{\prime}_i},{e^{\prime}_i})$ such as $k \in \,[ {{s_i},{e_i}} )$, which means that there is an overlap between the two vectors, we assign the motif / region pair to one of the two following categories: If ${r_i} = \ r_i^{\prime}$ we consider them to have the same strand orientation, if ${r_i} \ne r_i^{\prime}$ we consider them to have the opposite orientation. Using the total number of pairs in same strand orientation and opposite strand orientation, we calculate the strand asymmetry ratio as follows:

$$\begin{eqnarray*} && Same\ /\ Opposite\ Strand\ Asymmetry\ Ratio \nonumber \\ && = \frac{{Occurrences\ in\ same\ strand\ orientation}}{{Occurrences\ in\ same + opposite\ strand\ orientation}} \end{eqnarray*}$$

We symbolize the number of occurrences in same strand orientation as *k* and occurrences in opposite strand orientation as *l*. We define $T\ = \ k + l$ as the total number of comparisons. We then calculate the *P*-value for the two-tailed binomial test as follows, where *P* is the user-defined probability for same strand orientation (default = 0.5, assuming a random distribution of the orientation between motifs and regions).

$$\begin{eqnarray*} p - value &=& \mathop \sum \limits_{i = 0}^k C\left( {T,i} \right) \times {p^i} \times {q^{T - i}} \nonumber \\ && + \mathop \sum \limits_{i = T - k}^T C\left( {T,i} \right) \times {p^i} \times {q^{T - i}},\ q = 1 - p\end{eqnarray*}$$

The corresponding *P*-values are calculated using the ‘scipy’ package in Python (24) and are adjusted with Bonferroni correction in case of multiple tests.

The outputs include a table with the strand asymmetry score and statistics for each comparison. It also includes visualizations in the form of barplots for the number of occurrences in same versus opposite orientations and other relevant statistics.

### Strand asymmetry estimation between pairs of proximal motifs

The tool uses as input a pair of BED files representing two motifs. Let *S_1_,S_2_* be the set representation of two strand-annotated BED files. For each vector ${v_i} = ({c_i},{s_i},{e_i},{r_i})$ in *S_1_* we use the ‘bedtools closest’ function to determine the closest element in *S_2_*, ${v^{\prime}_i} = ({c^{\prime}_i},{s^{\prime}_i},{e^{\prime}_i},{r^{\prime}_i})$, such as $\forall v_i^{^{\prime\prime}} \in {S_2}:\ d( {{v_i},\ v_i^{^{\prime\prime}}} ) \ge \ d( {{v_i},v_i^{\prime}} )$. In the case of a tie, i.e. multiple $v_i^{^{\prime\prime}}$ fulfilling that criterion, all instances are reported by default and are considered for the subsequent analysis. If the distance between the two is within the user-specified parameters, then the pair is assigned to the following categories; If ${r_i} = r_i^{\prime}$, then they are considered to have the same orientation (Figure 2C). Conversely, if ${r_i} \ne r_i^{\prime}$ they are considered to have the opposite orientation. If they have the opposite orientation, there is a further subdivision in convergent or divergent (Figure 2D). Let ${v_k} = {v_i}$ if ${s_i} \le s_i^{\prime}$ and ${v_k} = v_i^{\prime}$ otherwise. If ${r_k} = \ +$ then the pair is considered convergent, otherwise it is considered divergent (Figure 2E).

Strand asymmetry ratios are calculated as:

$$\begin{eqnarray*} && Same\ /\ Opposite\ Strand\ Asymmetry\ Ratio \nonumber \\ && = \frac{{Occurrences\ in\ same\ strand\ orientation}}{{Occurrences\ in\ same + opposite\ strand\ orientation}} \end{eqnarray*}$$

$$\begin{eqnarray*} && Convergent\ /\ Divergent\ Asymmetry\ Ratio \nonumber \\ && = \frac{{Occurrences\ in\ convergent\ orientation}}{{Occurrences\ in\ convergent + divergent\ orientation}} \end{eqnarray*}$$

To calculate the corresponding *P*-values the same procedure is followed as described in the methods section of strand asymmetry estimation between regions and overlapping motifs. The convergent/divergent *P*-value is calculated similarly to the same/opposite *P*-value, with *k* the number of occurrences in convergent strand orientation and *l* the number of occurrences in divergent strand orientation.

The outputs include a table of the asymmetries for same versus opposite strand and convergent versus divergent orientations. It also includes barplots for each asymmetry comparison, distribution plots showing the strand asymmetries as a function of distance between motif pairs and other relevant statistics.

### Orientation assignment prior to asymmetry analysis

The previous functions are based on the fact that the motifs are assigned a specific strand (+ or −) because they are found either on *A_n_* or *B_n_*. In the case that the feature of one file is present in both strands and thus lacks strand annotation, it is possible to assign to it a strand annotation based on a feature provided in a second file. For this, the user needs to provide an un-annotated BED file, as well as one annotated BED file of a different feature using the same genome annotation. Let *S_1_, S_2_* be the sets representing the two BED files, with *S_1_* representing the annotated file *S_2_* the un-annotated file. For each vector ${v_i} = ({c_i},{s_i},{e_i},{r_i})$ in *S_2_*, this function will compare it to every vector ${v^{\prime}_i} = ({c^{\prime}_i},{s^{\prime}_i},{e^{\prime}_i},{r^{\prime}_i})$, in *S_1_*. If $\exists k \in [{s^{\prime}_i},{e^{\prime}_i})$ such as $k \in \,[ {{s_i},{e_i}} )$, which means that there is an overlap between the two vectors, then we set ${r_i} = r_i^{\prime}$,. If there are multiple vectors that fulfil the criteria of $v_i^{\prime}$ then only the one with the minimal distance between the centers of ${v_i}$ and $v_i^{\prime}$ ,defined as $| {({s_i} + {e_i})/2 - ( {{{s^{\prime}}_i} + {{e^{\prime}}_i}} )/2} |$ is kept.

### Genomic analyses

The human genome built hg38 was used throughout this work. Gene annotation from GENCODE was used (v33); the file was derived from (https://www.gencodegenes.org/) and filtered to include only protein-coding genes (25). Germline structural variant data were downloaded from the gnomAD (v2) website (https://gnomad.broadinstitute.org/), with version 2 of the database being used (26). Coordinates of transposable elements were derived for the human genome (hg38) from the http://hgdownload.soe.ucsc.edu/goldenPath/hg38/database/rmsk.txt.gz (version from 11 March 2019) which uses RepeatMasker (Smit Hubley Green, www.repeatmasker.org) and were filtered to include LINE, SINE and LTR transposable elements. Repli-seq data for the BG02ES cell line were obtained from the ENCODE project (Release 2) (https://www.encodeproject.org/) (27) and lifted over to hg38; leading and lagging orientation of the replication machinery across the human genome was inferred as described in (13). Genes were divided into ten equal-sized bins, with an upstream and a downstream 1kB bin added for each gene, resulting in twelve bins. Pearson correlations between transcriptional strand asymmetry of transposable elements and bin number was performed excluding the upstream and downstream 1kB bins. Position frequency matrices (PMWs) of transcription factors were derived from JASPAR (release 2020) for the non-redundant CORE collection (28) (http://jaspar.genereg.net/download/CORE/JASPAR2020_CORE_vertebrates_non-redundant_pfms_meme.zip) and motif scanning was performed with FIMO using as background model the nucleotide frequencies across the human genome and requiring a minimum *P*-value <10^−6^ (29). Transcription factors for which no motif occurrences below the *P*-value threshold in the human genome, were excluded from the analyses. Unibind PWM motif maps (https://unibind.uio.no/static/data/bulk/pwm_tfbs_per_tf.tar.gz), from the 2019 release, extracted from ChIP-seq experiments of their corresponding transcription factor with peak-caller MACS were analysed for transcriptional strand asymmetry across genic regions (30). Statistical analysis was performed in Python with the packages ‘math’, ‘scipy’, ‘pandas’ and ‘numpy’ and in R; visualizations were performed in Python with ‘matplotlib’ and ‘seaborn’ packages and in R with the ‘ggplot2’ package.

### Estimation of endogenous repeat element asymmetries

Transcriptional and replicative strand asymmetries of endogenous repeat elements were estimated as:

$$\begin{eqnarray*} && Background\ Transcriptional\ Strand\ Asymmetry\ Ratio \nonumber \\ && = \frac{{Occurrences\ of\ repeat\ in\ non - template\ strand\ }}{{Occurrences\ of\ repeat\ in\ template\ and\ non - template\ strand}} \end{eqnarray*}$$

$$\begin{eqnarray*} && Background\ Replicative\ Strand\ Asymmetry\ Ratio \nonumber \\ && = \frac{{Occurrences\ of\ repeat\ in\ leading\ strand\ orientation\ }}{{Occurrences\ of\ repeat\ in\ leading\ and\ lagging\ strand\ orientation}}\end{eqnarray*}$$

To calculate the corresponding Bonferroni-corrected *P*-values the same procedure is followed as described in the methods section of strand asymmetry estimation between regions and overlapping motifs.

When calculating the bias in breakpoints in template/non-template and leading/lagging strands to correct for background asymmetries in the orientation of endogenous repeat elements we estimated the adjusted strand asymmetry ratio as:

$$\begin{eqnarray*} && Observed\ Transcriptional\ Strand\ Asymmetry\ Ratio \nonumber \\ && = \frac{{Breakpoints\ in\ repeats\ in\ non - template\ strand\ orientation}}{{Breakpoints\ in\ repeats\ in\ template\ and\ non - template\ strand\ orientation}}\end{eqnarray*}$$

$$\begin{eqnarray*} && Observed\ Replicative\ Strand\ Asymmetry\ Ratio \nonumber \\ && = \frac{{Breakpoints\ in\ repeats\ in\ leading\ orientation}}{{Breakpoints\ in\ repeats\ in\ leading\ and\ lagging\ strand\ orientation}}\end{eqnarray*}$$

From which we calculated the adjusted strand asymmetry ratio for transcriptional and replicative strand asymmetries for the breakpoints:

$$\begin{eqnarray*} && Adjusted\ Transcriptional\ Strand\ Asymmetry\ Ratio \nonumber \\ && = \frac{{Observed\ Transcriptional\ Strand\ Asymmetry\ Ratio\ }}{{Background\ Transcriptional\ Strand\ Asymmetry\ Ratio}}\end{eqnarray*}$$

$$\begin{eqnarray*} && Adjusted\ Replicative\ Strand\ Asymmetry\ Ratio \nonumber \\ && = \frac{{Observed\ Replicative\ Strand\ Asymmetry\ Ratio\ }}{{Background\ Replicative\ Strand\ Asymmetry\ Ratio}}\end{eqnarray*}$$

We then calculate the Bonferroni-corrected *P*-values, as described in the methods section, replacing the expected binomial probability *P* with the probability of background transcriptional strand asymmetry and the background replicative strand asymmetry of each endogenous repeat element respectively.

### TFBS transcriptional strand asymmetry estimation

For each transcription factor the transcriptional strand asymmetry of its TFBSs was estimated as:

$$\begin{eqnarray*} && Transcriptional\ Strand\ Asymmetry\ Ratio \nonumber \\ && = \frac{{Occurrences\ of\ TFBS\ in\ non - template\ strand\ }}{{Occurrences\ of\ TFBS\ in\ template\ and\ non - template\ strand}}\end{eqnarray*}$$

To calculate the corresponding Bonferroni-corrected *P*-values the same procedure is followed as described in the methods section of strand asymmetry estimation between regions and overlapping motifs.

For each PWM motif of each transcription factor we generated simulations (*N* = 100) in which we randomly shuffled the order of the rows. For each of these simulated PWMs we generated genome-wide maps of their motif occurrences. Next, we estimated the expected transcriptional strand asymmetry ratio of each simulated PWM and calculated the mean transcriptional strand asymmetry ratio across all simulations, resulting in an expected transcriptional strand asymmetry. The adjusted transcriptional strand asymmetry ratio was estimated as the transcriptional strand asymmetry ratio of the original PWM over the mean expected transcriptional strand asymmetry ratio from the simulations. We then calculate the Bonferroni-corrected *P*-value, as described in the methods section, replacing the default probability of same strand orientation with the mean probability of the simulations.

## RESULTS

To illustrate the use of Asymmetron, we carried out analyses, which resulted in novel biological insights: (i) by orienting germline structural variant breakpoints relative to transposable elements we identify transcriptional and replicative strand asymmetries; (ii) we provide evidence that the orientation of a large portion of TFBSs is biased relative to the transcription direction, across the human transcriptome; (iii) we show that closely-spaced homotypic CTCF binding sites are more likely to be in the same orientation; (iv) in addition, we show how to use Asymmetron to detect motif strand asymmetries by using a previously characterized motif bias between the TATA-box and the initiator element.

### Transposable element orientations reveal structural variant asymmetries

Although strand asymmetries for nucleotide substitutions, insertions and deletions (indels) have been previously characterized using the trinucleotide context of substitutions or the repetitive patterns at the site of indels (13,14,31) a strand asymmetry analysis has not been performed for structural variants. Here, we investigated transcriptional and replicative strand asymmetries of three transposable elements, Long Interspersed Nuclear Elements (LINEs), Short Interspersed Nuclear Elements (SINEs) and Long Terminal Repeats (LTRs). We also oriented population structural variant breakpoints from the Genome Aggregation Database (gnomAD) (26) for the likelihood to occur at a particular orientation at each of these repetitive elements.

We first analyzed the transcriptional strand asymmetry of each of these types of repetitive elements across genic regions using Asymmetron:

python contained_asymmetries.py gencode.v33.annotation.bed LINEs.bed

**Figure 3.**
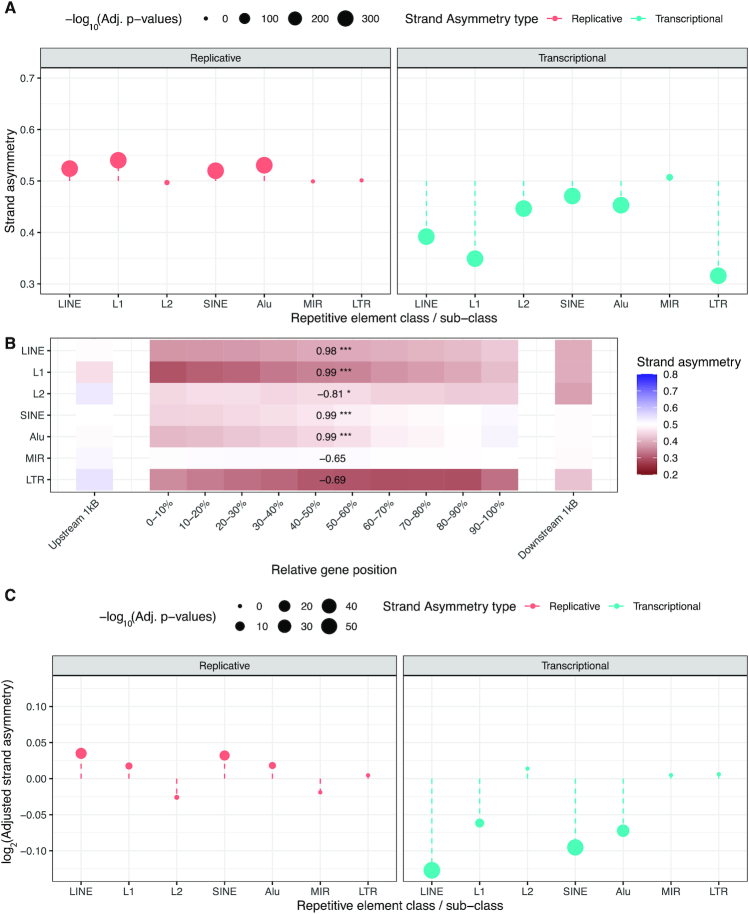
Transcriptional and replicative strand asymmetries of structural variants using transposable elements for their orientation. (**A**) Transcriptional and replicative strand asymmetry bias for endogenous retroelements. Adjusted *P*-values are Bonferroni corrected and are derived from binomial tests. Transcriptional strand asymmetry is the ratio of non-template to non-template and template occurrences, while replicative strand asymmetry is the ratio of leading orientation occurrences to leading and lagging occurrences of a transposable element. (**B**) Transcriptional strand asymmetry bias of endogenous retroelements relative to their position across the gene. Pearson correlations were estimated for the ten bins between the TSS and the TES. Adjusted *P*-values displayed as * for *P*-value <0.05, ** for *P*-value <0.01 and *** for *P*-value <0.001. (**C**) Log_2_ adjusted strand asymmetry ratio for transcriptional and replicative strand asymmetry of structural variant breakpoints overlapping endogenous retroelements correcting for their background strand asymmetries. Adjusted *P*-values are Bonferroni corrected and are derived from binomial tests.

The strand asymmetry was measured as the ratio of non-template to total occurrences. A ratio above 0.5 reflects a preference of the transposable element for the non-template strand, while a ratio below 0.5 reflects a bias towards the template strand orientation. We found a preference for LINEs, SINEs and LTRs to be at the template strand (ratios 0.392, 0.471, 0.316, respectively, binomial test, Bonferroni corrected *P*-values < 0.001) (Figure 3A), consistent with previous reports (32–34). We also subdivided SINEs into Alu repeats and Mammalian-wide interspersed repeats (MIRs), finding strong transcriptional strand asymmetries only in Alu repeats (Figure 3A). Similarly, we divided LINE retrotransposons in L1 and L2 and found significant transcriptional strand asymmetries in both, which were more pronounced for L1 repeats (Figure 3A).

We investigated the transcriptional strand asymmetry of transposable elements as a function of their position in the gene. To perform this, we separated each gene into ten equal-sized bins and added a 1 kB upstream window bin and a 1 kB downstream window bin (Figure 3B). For LINEs and SINEs we found a more pronounced template strand asymmetry bias in regions closer to the TSS, which decreased as a function of distance from it, whereas for LTRs, we could not observe a statistically significant correlation (Figure 3B). We also observed a positive correlation with relative position in the gene and transcriptional strand asymmetry for Alu and L1 repeats, whereas MIR repeats did not display a statistically significant correlation and L2 elements displayed a negative correlation (Figure 3B). These results suggest that transposable elements are preferentially located relative to orientation and position in genic regions.

Next, we investigated the frequency of structural variants at each of these elements at the template and non-template strand orientations. To perform this analysis, we oriented structural variants relative to endogenous elements:

python orientation.py gnomad_v2.1_sv.sites.bed LINEs.bed

After correcting for the background asymmetries of their orientation preferences within transcribed regions, we observed that for LINEs and SINEs there was a significant association between their orientation and the probability of harboring a structural variant breakpoint, with a preference for the template strand (Adjusted transcriptional strand asymmetry ratios of 0.916 and 0.936; binomial test, Bonferroni corrected, *P*-value < 0.001), while for LTRs we could not find a preference (Figure 3C). When we subdivided LINE and SINE repeat elements, we found that the structural variant breakpoint transcriptional strand asymmetries were found for L1 and Alu repeat elements (binomial test, Bonferroni corrected, *P*-value < 0.001), but not for L2 or MIR elements (*P*-value > 0.05) (Figure 3C).

python contained_asymmetries.py gencode.v33.annotation.bed gnomad_v2.1_sv.sites.LINEs.bed –expected_asym = 0.392

Next, we investigated if the directionality of the replication fork was associated with the orientation of LINEs, SINEs and LTRs and if their orientation also influences the likelihood of observing structural variant breakpoints within those elements. We used Repli-seq data from BG02ES (27), a human embryonic stem cell (ESC) line, to infer the directionality of the replication-fork genome-wide. Similarly to the transcriptional strand asymmetry ratio, the replicative strand asymmetry ratio reflected the occurrences of the transposable elements in the leading orientation over their total occurrences. We found that LINEs and SINEs were more likely to be found in the leading strand orientation (Strand asymmetry: LINEs: 0.524, SINEs: 0.520, binomial test, Bonferroni corrected *P*-values < 0.001), whereas LTRs did not display a significant orientation bias (*P*-value > 0.05) (Figure 3A). For SINEs, we found replicative strand asymmetries at Alu repeats but not at MIR repeats (Figure 3A). We also separated LINEs into L1 and L2 repeats and found replicative strand asymmetries only for L1 repeats (Figure 3A).

python contained_asymmetries.py Bg02es_RepliStrand.bed LINEs.bed

We investigated if the replicative orientation of these endogenous elements was associated with the likelihood of observing germline structural variants. To perform this, we used the structural variant breakpoints that were oriented relative to the repetitive elements and studied their replicative strand asymmetry:

python orientation.py Bg02es_RepliStrand.bed LINEs.bed

We corrected for the background asymmetry in the orientation of each transposable element and investigated if structural variant breakpoints were more likely to be found at a specific orientation. We found that for both LINEs and SINEs there was a significant strand asymmetry with a higher frequency of structural variant breakpoints at the leading orientation (*P*-value < 0.001), whereas for LTRs no bias was detected (*P*-value > 0.05), (Figure 3C). We performed the same analysis for LINE and SINE repeat elements and found that the structural variant breakpoint replicative strand asymmetry was found for L1 and Alu elements, but not for L2 or MIR elements (Figure 3C), similar to our results regarding the transcriptional strand asymmetries.

python contained_asymmetries.py Bg02es_RepliStrand.bed LINEs.bed gnomad_v2.1_sv.sites.LINEs.bed –expected_asym = 0.524

We also separated Alu repeats in the three subfamilies (AluJ, AluY and AluS) and L1 repeats in primate-specific (L1P) and mammalian-wide (L1M) and found consistent transcriptional and replicative strand asymmetries in all of them (Supplementary Table S1). However, we only found breakpoint strand asymmetries in AluY and AluS repeats for Alu subfamilies and L1P for L1 subfamilies (Supplementary Table S2), consistent with previous work indicating that only members of AluY, AluS and L1P subfamilies remain active in the human genome (35,36). However, we currently cannot rule out the contribution of other mechanisms such as nonallelic homologous recombination contributing to the observed differences at younger repeats and future experimental work is required to provide additional evidence for this.

Finally, we investigated if the transcriptional and replicative strand asymmetries of transposable elements were dependent on each other or if they were independent contributors. When we controlled for transcription direction and performed the replicative strand asymmetry analysis, the results remained largely unaltered, as was the case when controlling replicative orientation and performing the transcriptional strand asymmetry analysis. These results provide additional evidence that endogenous repeat elements have orientation preferences determined by both replication and transcription.

### Strand asymmetries of TFBSs at promoters and across transcribed regions

Many regulatory elements are found within transcribed regions. Nevertheless, it remains unknown if the transcription process influences the transcription factor DNA strand regulatory grammar within transcribed regions. Here, we generated a transcriptome-wide map of human TFBSs with FIMO (29) using the JASPAR vertebrate non-redundant list of transcription factors (28). We filtered out transcription factor Position Weight Matrices (PWMs) for which there were no matches meeting the *P*-value threshold, resulting in 551 PWMs, representing a diverse set of transcription factors. We oriented each TFBS occurrence with respect to the transcription direction as template or non-template. As a null hypothesis, we assumed that TFBSs are equally likely to occur at both orientations.

**Figure 4.**
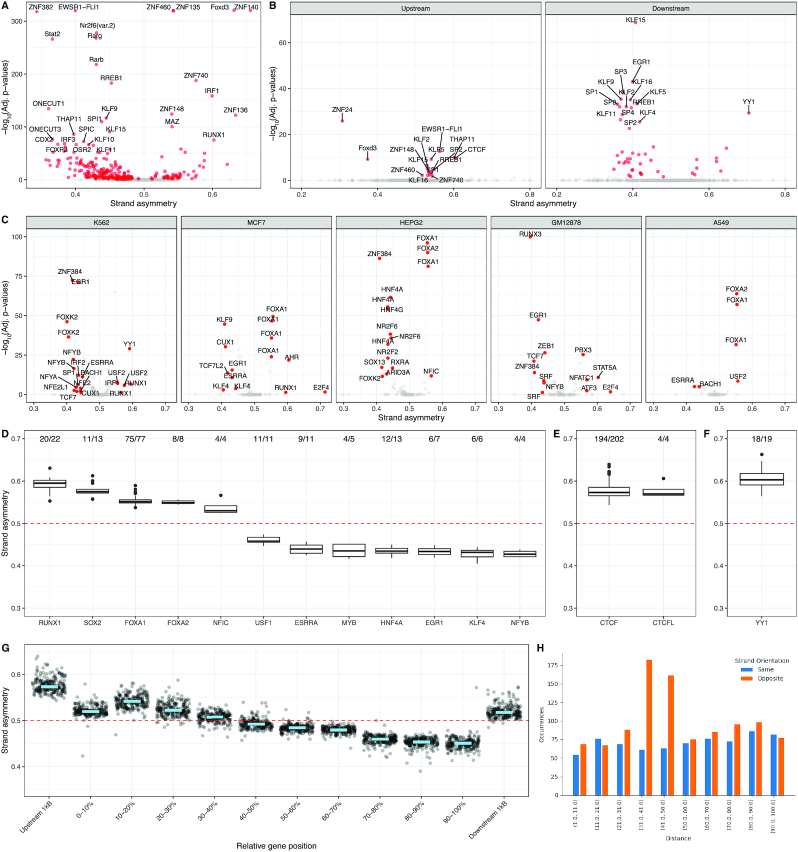
A large proportion of transcription factor binding sites display significant transcriptional strand asymmetry bias. (A, B) Volcano plots showing the transcriptional strand asymmetry of TFBSs and the associated *P*-values from binomial testing with Bonferroni correction, for multiple transcription factors. Grey colored marks represent TFBSs with non-statistically significant strand asymmetries. (**A**) Across transcribed regions. (**B**) Across 1 kB upstream from the TSS and across 1kB downstream from the TSS (**C**) Volcano plots across five cell lines showing the strand asymmetry and Bonferroni corrected binomial *P*-values of TFBSs for each ChIP-seq experiment. Strand asymmetry relative to gene orientation of transcription factors found in at least four ChIP-seq experiments and showing statistically significant TFBS strand asymmetry bias for at least 75% of the experiments performed. (D−F) Box plots display the statistically significant strand asymmetry scores for each transcription factor across ChIP-seq experiments. (**D**) Strand asymmetry across transcribed regions. (**E**) Strand asymmetry across promoter upstream regions. (**F**) Strand asymmetry across promoter downstream regions. The embedded text in D-F displays the number of ChIP-seq experiments for which statistically significant TFBS strand asymmetry was observed. (**G**) CTCF motif orientation across genic regions, separated in bins. Each dot represents a ChIP-seq experiment for which the CTCF motif with the highest binding score and proximity to the center of each peak were used. The light blue line represents the median strand asymmetry across all ChIP-seq experiments. (**H**) Strand asymmetry analysis of TATA-box and INR motifs as a function of their pairwise distance within promoter regions.

First, we investigated if the TFBS orientation biases could be identified across transcribed regions (transcription start to transcription end). We found that out of 551 TF PWMs, 248 (45%) displayed significant transcriptional strand asymmetries (binomial tests with *P*-value < 0.05, Bonferroni corrected) (Figure 4A). To account for the influence of the nucleotide composition in TFBS transcriptional strand asymmetries, we shuffled the order of the rows of each PWM 100 times, from which we estimated the average expected transcriptional strand asymmetries. After correcting for nucleotide composition biases, we found 150 (27%) of transcription factors showed significant transcriptional strand asymmetries, with 73% being shared with our earlier model (Supplementary Figure S1a). These results indicate that the orientation of TFBSs is not random across transcribed regions.

We also compared the strand asymmetry bias of each TFBS in the promoter upstream region (−1000 bp to Transcription Start Site (TSS)) and the promoter downstream region (TSS to 1000 bp). We found that on average TFBSs displayed stronger strand asymmetry patterns in the downstream promoter regions; with median absolute orientation biases of 7.14% versus 11.25% in the upstream and downstream promoter regions (Mann−Whitney *U*, *P*-value = 3.1e−9), (Figure 4B). However, the stronger strand asymmetry patterns for the downstream promoter region relative to the upstream promoter regions were explained by the nucleotide composition restrictions of the first (Supplementary Figure S1b). These results are in accordance with the notion that the transcription process imposes restrictions in the orientation preference of TFBSs.

An example of the Asymmetron command for one of the transcription factors:

python contained_asymmetries.py gencode.v33.1kB_upstream.bed CTCF.bed
python contained_asymmetries.py gencode.v33.1kB_downstream.bed CTCF.bed

To provide additional evidence that the observed TFBS strand asymmetries relative to the transcription direction reflect differences in the likelihood of transcription factor binding, we performed an extended analysis using the UniBind dataset. This dataset encompasses ChIP-seq experiments of 231 transcription regulators studied across 315 diverse cell lines and conditions (30). For each ChIP-seq peak in each experiment, the TFBS with the highest binding score and closest proximity to the peak summit for the corresponding transcription factor is selected, generating genome-wide high confidence TFBS maps. Using this dataset, we compared the strand asymmetry of TFBSs upstream and downstream of the TSS.

We measured the orientation preference of each transcription factor across cell lines and conditions at transcribed regions and found that transcription factors displayed significant orientation preference relative to the transcription direction in ∼20% of ChIP-seq experiments (binomial test with Bonferroni correction, *P*-value < 0.05). We focused our analysis on the five cell lines with the most experiments available, K562, MCF7, HEPG2, GM12878 and A549. We found that certain transcription factors consistently displayed orientation preference across multiple experiments and across different cell lines (Figure 4C). Some of the most pronounced asymmetries included those of RUNX1, SOX2, FOXA1, FOXA2, ZNF384, HNF4A, HNF4G, EGR1, ESRRA, NFYA, NFYB, NFIC, USF1, USF2, E2F4 and KLF4. When we compared ChIP-seq experiments across cell lines for these transcription factors, we found consistency in both the orientation preference and the statistical significance (Figure 4C and D).

We also subdivided the analysis in the promoter upstream region (−1000 bp to TSS) and the promoter downstream region (TSS to 1000 bp). Although we were underpowered, we found that in the promoter upstream region CTCF and CTCFL TFBSs consistently displayed a preference for the non-template strand. In particular, out of 202 ChIP-seq experiments analyzed, 194 of them showed statistically significant orientation preference of the CTCF motif for the non-template strand after multiple testing correction in the promoter upstream region (Figure 4E). In the promoter downstream region, YY1 displayed a preference for the non-template strand with a statistically significant strand asymmetry in 18 out of 19 experiments analyzed (Figure 4F). For CTCF, we investigated if the observed transcriptional strand asymmetry was influenced by its motif positioning across the gene. We found across ChIP-seq experiments that the bound CTCF motif orientations were influenced by the position in the gene, with a negative correlation relative to bin number from TSS to Transcription End Site (TES) (Pearson correlation, *r* = −0.96, *P*-value < 0.001) and a non-template strand asymmetry in promoter upstream and transcription termination downstream regions (Figure 4G). These results confirm our previous findings which showed that transcription factors are not strand agnostic and that orientation relative to the TSS could determine function.

### Detecting motif orientation bias in homotypic motif occurrences

To showcase how Asymmetron can be used to study the orientation preference of consecutive occurrences of a motif we performed a case study on the CTCF motif. We investigated the orientation bias of high confidence homotypic CTCF motif occurrences across the human genome. We found a preference for the same orientation for consecutive CTCF motif occurrences within distances of up to 100bp (empirical *P*-value < 0.001).

python consecutive_patterns.py CTCF.bed

Other potential implementations of this function could include the identification of miRNA clusters with strand bias, investigation of CTCF orientations at long genomic distances and 3D organization of the genome (same / opposite, convergent/divergent orientation analyses) or the orientation preferences of endogenous repeat elements among others.

### Detecting motif orientation bias in motif pairs using Asymmetron

To show how to implement Asymmetron to study strand asymmetries in motif pairs, we used a previously characterized orientation bias of the TATA-box relative to the initiator element (INR) (37), the locations of both of which were extracted using JASPAR PWM motifs. We focused our analysis at regions around the TSS (-1,000bp to +1,000bp). We found that both the orientation and the pairwise distance of the two motifs was highly biased (Figure 4H) and consistent with the literature (38). In particular, there was a significant bias relative to their orientation with preference for the opposite strand (*P*-value < e−11), which was pronounced at a pairwise distance of 30–50 bp and which disappeared for shorter or longer distances (Figure 4H).

python pairwise_asymmetries.py TATA_box.bed INR.bed

## DISCUSSION

Asymmetron is a multi-purpose toolkit that enables the exploration of strand asymmetries in diverse biological problems. We applied Asymmetron to four different biological problems showing that: (i) germline structural variants are more likely to overlap LINE and SINE transposable elements with particular orientations relative to transcription and replication direction, (ii) 45% of transcription factors show highly biased TFBS orientation preferences relative to transcriptional direction across human genic regions, (iii) orientation bias in homotypic occurrences of nearby CTCF motifs towards the same strand and (iv) motif orientation bias for TATA-box and INR motifs found at the core promoter.

The observed background asymmetries in the orientation of LINE and SINE transposable elements could have been influenced by the contribution of polyadenylation signals within them through selection pressure, as previously suggested (33). In addition, dynamic changes in replication directionality during evolution could explain the weaker strand asymmetry biases observed, relative to transcriptional strand asymmetry biases, especially for inactive transposable elements (Figure 3A). The identification of biases in population structural variant breakpoints relative to LINE and SINE transposable elements suggests activity of these elements in the germline, which has been influenced by their orientation relative to the direction of replication and transcription. MIR and L2 repeats have lost the ability to retrotranspose (34), whereas a small subset of L1 and Alu repeats remain active today (35,36,39,40). The observed strand asymmetries at L1 and Alu repeats are consistent with this notion and with previous work, finding a preference of L1 repeat integration towards the leading orientation (41). The absence of structural variant strand asymmetries at LTRs is also consistent with the notion that these elements are inactive. Additional work is required to understand if the observed asymmetries of structural variants at transposable elements are aggravated in cancer and other disorders and if they are associated with disease development.

We have shown that the orientation preference of multiple TFBSs around promoters and at transcribed regions cannot be explained by the nucleotide composition differences in the template and non-template strands (Supplementary Figure S1). Therefore, it could be the result of transcription factor preferential affinity for the motif at the forward or reverse-complement orientations and interaction or interference with RNA polymerase progression. A strand preference could also indicate roles of certain transcription factors at the RNA level, examples being SOX2 (Figure 4E) and YY1 (Figure 4F), which are known to bind both DNA and RNA to regulate gene expression (42,43). Our results suggest that TFBS orientation in transcribed regions is non-random and influences gene regulatory grammar. However, it remains unknown how the orientation of transcription factors between closely spaced TFBSs influences steric hindrance and competition for binding (44). Experimental designs that systematically evaluate how the orientation of TFBSs within *cis*-regulatory modules influence regulatory element activity could further increase our understanding. The conglomeration of transcription factors in *cis*-regulatory modules could be influenced by the orientation and pairwise distance of TFBSs and high-throughput reporter assay experiments (45) could provide valuable insights in this direction.

Asymmetron enables the study of asymmetric biological processes. Investigation of transcriptional and replicative strand asymmetries across biological organisms reflects the number of replication forks and their orientation, the gene density and the diverse mechanisms safeguarding genome integrity (1–4). Strand asymmetry analysis could increase our understanding of mutational processes across different disorders and evolution (6,13). In cancer, the orientation of structural variant breakpoints could reveal unknown mutational mechanisms. In gene regulation, investigation of orientation preferences between transcription factors and their location relative to transcriptional direction could enable better modelling of gene expression. In summary, we have shown that Asymmetron can pose as a useful tool to annotate and detect DNA strand asymmetries and associate them with specific biological functions.

## DATA AVAILABILITY

A Python implementation package can be found in GitHub:

https://github.com/Ahituv-lab/Asymmetron.

## Supplementary Material

gkaa1052_Supplemental_File
