# Supplementary material for: Asymmetron: a toolkit for the identification of strand asymmetry patterns in biological sequences
Source: Nucleic Acids Res. 2020 Nov 19;49(1):e4. doi: 10.1093/nar/gkaa1052 (PMC7797064; doi:10.1093/nar/gkaa1052)
Supplement: gkaa1052_Supplemental_File [file gkaa1052_supplemental_file.pdf]

Supplementary Material

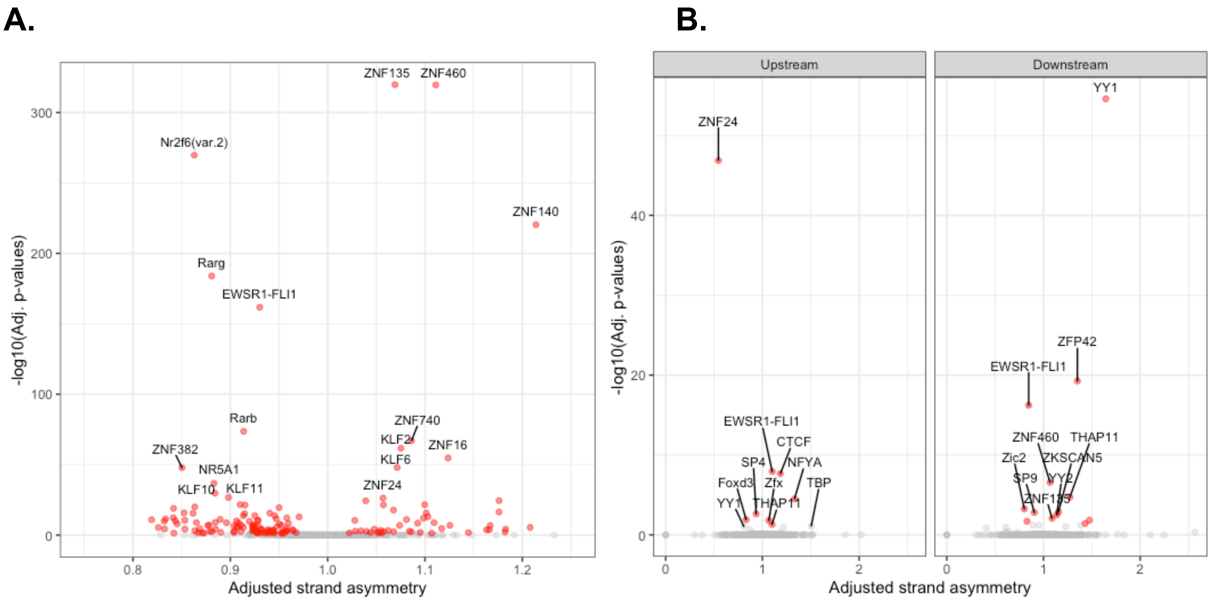

Supplementary Figure 1: A large proportion of transcription factor binding sites display significant transcriptional strand asymmetry bias after controlling for nucleotide composition of the PWM motifs. A-B. Volcano plots showing adjusted strand asymmetry ratios of the transcriptional strand asymmetry of TFBSs and the associated p-values from binomial testing with Bonferroni correction, for multiple transcription factors. For each transcription factor, the adjusted strand asymmetry ratio was calculated from the transcriptional strand asymmetry of its corresponding TFBSs as identified using its corresponding PWM and the transcriptional strand asymmetry of the shuffled control PWMs.

Supplementary Table 1: Transcriptional and replicative strand asymmetries of Alu and L1 repeat subfamilies. Transcriptional and replicative strand asymmetry ratios are calculated as the occurrences in one orientation (leading or non-template) over the total number of occurrences in both orientations. Ratios above 0.5 indicate non-template and leading orientation preference, while ratios below 0.5 indicate template and lagging orientation preference for **t**ranscriptional and replicative strand asymmetries. Displayed p-values are from binomial testing with Bonferroni correction.

Repeat Subfamily	Repeat transcriptional strand asymmetry ratio	Repeat transcriptional strand asymmetry p-value	Repeat replicative strand asymmetry ratio	Repeat replicative strand asymmetry p-value
AluJ	0.472	<0.001	0.534	<0.001
AluS	0.449	<0.001	0.531	<0.001
AluY	0.459	<0.001	0.525	<0.001
L1M	0.373	<0.001	0.542	<0.001
L1P	0.409	<0.001	0.523	<0.001

Supplementary Table 2: Breakpoint transcriptional and replicative strand asymmetries of Alu and L1 repeat subfamilies. Adjusted transcriptional and replicative strand asymmetry ratios for breakpoints at repeats are displayed. Log2(adjusted strand asymmetry ratios) above 0 indicate non-template and leading orientation preference, while log2(adjusted strand asymmetry ratios) below 0 indicate template and lagging orientation preference for the breakpoints at Alu or L1 repeats. Displayed p-values are from binomial testing with Bonferroni correction.

Repeat Subfamily	Breakpoint transcriptional strand asymmetry log2(adjusted strand asymmetry ratios)	Breakpoint transcriptional strand asymmetry p-value	Breakpoint replicative strand asymmetry log2(adjusted strand asymmetry ratios)	Breakpoint replicative strand asymmetry p-value
AluJ	-0.040	>0.05	0.017	>0.05
AluS	-0.056	<0.001	0.023	<0.001
AluY	-0.056	<0.001	0.014	>0.05
L1M	-0.008	>0.05	0.004	>0.05
L1P	-0.163	<0.001	0.063	<0.001
